# Requirements for confirmation of PCR-RFLP results of polymorphisms

**DOI:** 10.18502/ijrm.v17i3.4521

**Published:** 2019-05-29

##  Dear Editor

As clinical investigators and researchers exploring the role of insulin receptor gene (INSR) in polycystic ovary syndrome (PCOS), we wish to express our concern about the study by Tehrani *et al*. (1) that aimed to explore the correlation between INSR and adiponectin genes and PCOS. We are afraid that it might have technical flaws and take confusion and concerns to the readership.

First, in the Materials and Methods section the authors stated that this study was a cross-sectional study, while in the Conclusion section they called it a case-control study. It is really confusing. This reveals a lack of statistical understanding of the cross-sectional or case-control studies and reduces the power of the study.

Most critically, we are concerned about the performance of the polymerase chain reaction-restriction fragments length polymorphism analysis (PCR-RFLP). Quality control is a major issue in the application of PCR-RFLP studies, and for this reason we usually adopt some quality control procedures, including using positive and negative controls, repeating the genotyping analysis, and confirming the results with DNA sequencing (2, 3). However, in this study no quality control program was represented in the genotyping section.

A series of studies have investigated the different genotypes of INSR in PCOS and control populations. Nearly all the reports showed that the least frequent genotype of rs1799817 was TT in both control and PCOS groups (2, 4–13), while in this study the most frequent one was TT. Meanwhile, for rs2059806, all the other reports showed that the least frequent genotype was AA (2, 8, 12), while this study found that the most frequent one was AA. Additionally, a study from Iran was included in these studies, which showed that the genotype frequencies of CC/CT/TT of rs1799817 were 110/63/8 in control group and 105/64/12 in PCOS group (2), and the genotypes of GG/GA/AA of rs2059806 were 93/70/18 in control group and 96/73/12 in PCOS group (2). In this study, the genotypes of CC/CT/TT of rs1799817 were 7/54/95 in control population and 15/57/114 in PCOS population, and the genotypes of GG/GA/AA of rs2059806 were 18/58/80 in control and 11/76/99 in PCOS population. This distribution of genotypes is completely contradictory to the previously published one (2). Considering that they were carried out in the same ethnicity, it is difficult to explain it. This disaccordance raised a question about the molecular methods applied to define the genotypes. Further confirmation was required to make their results persuasive.

The authors also listed the allele frequencies in Table I. When we calculated the allele frequencies using the genotype frequencies provided by them, we obtained totally different results. For rs2059806 in PCOS, the frequency of allele A (FA) was calculated as FAA*2+FAG*1 = 99*2+76*1 = 274, and FG was calculated as FGG*2+FAG*1 = 11*2+76*1 = 98. However, the reported allele frequency of A was 110 and that of G was 76. Could the authors explain how they calculated the allele frequencies?

Finally, in Table IV, the haplotype of Insulin in PCOS included CA, CG, TA, and CG, which shows that a TG was likely mistaken as a CG, and therefore CG duplicated here. Similarly, the same problem happened in the haplotype of Insulin in controls too. In Figure 1, a TG might have been replaced by a CG too. What's more, the data labels of the haplotypes are not accurate either. There are three numbers without decimal places, including 7, 28, and 25, which should be 7.0, 28.0, and 25.8.

**Chun Feng${}^{1}$ M.D., Ping-ping Lv${}^{2}$ Ph.D. Candidate, Min Jin${}^{3}$ M.D., Ph.D., Jin-Ming Shen${}^{4}$ M.D., Tian-he Huang${}^{5}$ M.D.**

^1^Women's Hospital, School of Medicine, Zhejiang University, Hangzhou, Zhejiang, 310006, China.

^2^Key Laboratory of Reproductive Genetics, Ministry of Education (Zhejiang University), Hangzhou, Zhejiang, 310058, China.

^3^The Second Affiliated Hospital of Zhejiang University School of Medicine, Hangzhou, Zhejiang, 310009, China.

^4^The First Affiliated Hospital of Zhejiang Chinese Medicine University, Hangzhou, Zhejiang, 310018, China.

^5^The First Affiliated Hospital, College of Medicine of Xi'an Jiaotong University, Xi'an, Shanxi, 710061, China.

##  Authors' Answer

We thank Chun Feng and Colleagues for their interest in our paper (1). The issues raised (letter) concerning our study are important; this reply letter clarifies our methodology and corrects some results in detail.

A case-control study is a type of observational study in which two existing groups differing in outcomes are identified and compared on the basis of some supposed causal attribute, whereas a cross-sectional study provides a "snapshot" of a population at a single point in time and looks at both the diseases along with assumed covariates simultaneously (2). Our study was a case-control study in which a total of 186 women with PCOS using NIH criteria (cases) and 156 healthy women (controls) were recruited. At that time, we used the polymerase chain reaction-restriction fragments length polymorphism analysis (PCR-RFLP), and due to limited financial resources, we did not perform DNA sequencing; however, we used positive and negative control groups in each PCR. There was a typo error in the presentation of genotypes of INSR in our manuscript that created all the discrepancies between our results and those previously reported (3). We believe that the revised results presented in Table III will resolve the discrepancies. Results presented in this table should be replaced with the previous results presented in Table III of the original article and the corresponding texts in the Results section.

**Table 1:** Genotype, allele, and haplotype frequencies in the two groups of study


	**PCOS (n = 186)**	**Controls (n = 156)**
Genotype frequency (%)		
Adiponectin		
SmaI T > G (rs2241766)		
GG	2 (1.1)	4 (2.6)
TT	142 (76.3)	106 (67.9)
TG	42 (22.6)	46 (29.5)
BsmaI C > A (rs1501299)		
CC	92 (49.5)	77 (49.4)
AA	18 (9.7)	8 (5.1)
CA	76 (40.9)	71 (45.5)
Insulin		
Pm1I T > C (rs1799817)		
TT	114 (61.3)	95 (60.9)
CC	15 (8.1)	7 (4.5)
TC	57 (30.6)	54 (34.6)
NsiI A > G (rs2059806)		
AA	11 (5.9)	18 (11.5)
GG	99 (53.2)	80 (51.3)
AG	76 (40.9)	58 (37.2)
Allele frequency (%)		
SmaI T > G		
G	46	54
T	326	258
BsmaI C > A		
C	260	225
A	112	87
Pm1I T > C		
T	285	244
C	87	68
NsiI A > G		
A	98	94
G	274	218

**Fahimeh Ramezani Tehrani${}^{1}$ M.D., Maryam Daneshpour${}^{2}$ Ph.D., Somayeh Hashemi${}^{1}$ M.Sc., Maryam Zarkesh${}^{2}$ M.Sc., Feridoun Azizi${}^{3}$ M.D.**

^1^Reproductive Endocrinology Research Center, Research Institute for Endocrine Sciences, Shahid Beheshti University of Medical Sciences, Tehran, Iran.

^2^Obesity Research Center, Research Institute for Endocrine Sciences, Shahid Beheshti University of Medical Sciences, Tehran, Iran.

^3^Endocrine Research Center, Research Institute for Endocrine Sciences, Shahid Beheshti University of Medical Sciences, Tehran, Iran.

*Corresponding author:  Fahimeh Ramezani Tehrani;

Shahid Beheshti University of Medical Sciences, 24 Parvaneh, Yaman Street, Velenjak, Tehran, Iran. Postal Code: 19395-4763. email: ramezani@endocrine.ac.ir

Tel: (+98) 2122409309

##  Editorial

Thanks to Dr. Chun Feng and Colleagues for their comments on the article that was published in our journal issued 2013; 11: 185-194. The modified version of this article will be uploaded in our electronic version (March 2019).
